# Supplementary material for: A novel framework for educational Q&A: Leveraging RAG and Code Interpreters for knowledge retrieval and logical computation
Source: PLoS One. 2025 Dec 1;20(12):e0337361. doi: 10.1371/journal.pone.0337361 (PMC12668533; doi:10.1371/journal.pone.0337361)
Supplement: Attachment — Submitted filename: Response of Reviewer(Plos one).docx [file pone.0337361.s006.docx]

Journal Requirements:

When submitting your revision, we need you to address these additional requirements.

1. Please ensure that your manuscript meets PLOS ONE's style requirements, including those for file naming.

The PLOS ONE style templates can be found at

https://journals.plos.org/plosone/s/file?id=wjVg/PLOSOne_formatting_sample_main_body.pdf and

https://journals.plos.org/plosone/s/file?id=ba62/PLOSOne_formatting_sample_title_authors_affiliations.pdf

*A: After comparing the standardised documents of the journals, this article conforms to the style and naming convention of the article.*

2. Please note that PLOS ONE has specific guidelines on code sharing for submissions in which author-generated code underpins the findings in the manuscript. In these cases, we expect all author-generated code to be made available without restrictions upon publication of the work. Please review our guidelines at https://journals.plos.org/plosone/s/materials-and-software-sharing#loc-sharing-code and ensure that your code is shared in a way that follows best practice and facilitates reproducibility and reuse.

*A: The algorithms and simulation code covered in this paper are fully publicly available.*

3. Thank you for stating the following financial disclosure:

“This work is partially supported by 2025 Key Research Project of Shenzhen Polytechnic University "Research on Key Methods for Analysis and Prediction of Social Behaviour of Specific Characters on Multimodal Big Data (6025310008K)", 2024 Guangdong Province Education Science Planning Project (Higher Education Special Project) "Research on Smart Classroom Teaching Behavior Analysis Methods Based on Scene Semantic Understanding and Deep Learning Characteristic Representation (2024GXJK766)", 2023 Guangdong Provincial Higher Vocational Education Teaching Reform Research and Practice Project "Research on Online Teaching Quality Evaluation Method Based on Multimodal Affective State Analysis (2023JG277)", 2024 Shenzhen Polytechnic University Quality Engineering Project "Research on Classroom Scene Understanding and Behavior Analysis Method Based on Multimodal Attention Mechanisms (7024310268)", 2024 Higher Education Scientific Research Planning Project of the Chinese Society of Higher Education "Research on the Analysis of Teaching and Learning Deep Interaction Characteristics in Smart Classroom Environment Supported by Multimodal Data (24XH0407)", 2023 Shenzhen Education Science Planning Project"Research on the Evolutionary Mechanism and Intervention of Interpersonal Relationships among College Students Driven by Multimodal Data (rgzn23003)".

Please state what role the funders took in the study. If the funders had no role, please state: "The funders had no role in study design, data collection and analysis, decision to publish, or preparation of the manuscript."

If this statement is not correct you must amend it as needed.

Please include this amended Role of Funder statement in your cover letter; we will change the online submission form on your behalf.

*A: After careful combing, this paper mainly relies on the following six topics to carry out relevant research, and the algorithms proposed in the text are all supported by the following topics, including scientific research platforms, simulation data, and test applications.*

1. *2025 Guangdong Philosophy and Social Science Planning Project "Research on the Synergistic Evolutionary Mechanisms of the Governance System of Colleges and Universities and the Development of Students’ Socio-emotional Competence: Based on Multimodal Learning Analysis (GD25CJY29)".*
2. *2025 Key Research Project of Shenzhen Polytechnic University "Research on Key Methods for Analysis and Prediction of Social Behaviour of Specific Characters on Multimodal Big Data (6025310008K)", the research in this paper relies on this topic to carry out research aimed at solving the spatial mapping problem of multimodal heterogeneous features.*
3. *2024 Guangdong Province Education Science Planning Project (Higher Education Special Project) "Research on Smart Classroom Teaching Behavior Analysis Methods Based on Scene Semantic Understanding and Deep Learning Characteristic Representation (2024GXJK766)", based mainly on this topic, the research on behaviour detection is oriented towards the problem of joint mining for scene saliency regions and behaviour recognition.*
4. *2023 Guangdong Provincial Higher Vocational Education Teaching Reform Research and Practice Project "Research on Online Teaching Quality Evaluation Method Based on Multimodal Affective State Analysis (2023JG277)", based mainly on this topic, a behavioural prediction study for online teaching and learning is conducted by combining viewpoint summaries of teacher and student adaptive interaction behavioural events, propensity analysis and accurate modelling of online behavioural trajectories.*
5. *2024 Shenzhen Polytechnic University Quality Engineering Project "Research on Classroom Scene Understanding and Behavior Analysis Method Based on Multimodal Attention Mechanisms (7024310268)", in this study, a scene classification model based on the attention mechanism is proposed, theoretically validated and functionally tested in text.*
6. *2024 Higher Education Scientific Research Planning Project of the Chinese Society of Higher Education "Research on the Analysis of Teaching and Learning Deep Interaction Characteristics in Smart Classroom Environment Supported by Multimodal Data (24XH0407)", the definition and formal representation of the elements of teaching and learning activities in a smart classroom were investigated in that study based on automated quizzes, which were tested and validated in this paper.*

4. Thank you for stating in your Funding Statement:

“This work is partially supported by 2025 Key Research Project of Shenzhen Polytechnic University "Research on Key Methods for Analysis and Prediction of Social Behaviour of Specific Characters on Multimodal Big Data (6025310008K)", 2024 Guangdong Province Education Science Planning Project (Higher Education Special Project) "Research on Smart Classroom Teaching Behavior Analysis Methods Based on Scene Semantic Understanding and Deep Learning Characteristic Representation (2024GXJK766)", 2023 Guangdong Provincial Higher Vocational Education Teaching Reform Research and Practice Project "Research on Online Teaching Quality Evaluation Method Based on Multimodal Affective State Analysis (2023JG277)", 2024 Shenzhen Polytechnic University Quality Engineering Project "Research on Classroom Scene Understanding and Behavior Analysis Method Based on Multimodal Attention Mechanisms (7024310268)", 2024 Higher Education Scientific Research Planning Project of the Chinese Society of Higher Education "Research on the Analysis of Teaching and Learning Deep Interaction Characteristics in Smart Classroom Environment Supported by Multimodal Data (24XH0407)", 2023 Shenzhen Education Science Planning Project"Research on the Evolutionary Mechanism and Intervention of Interpersonal Relationships among College Students Driven by Multimodal Data (rgzn23003)".

Please provide an amended statement that declares *all* the funding or sources of support (whether external or internal to your organization) received during this study, as detailed online in our guide for authors at http://journals.plos.org/plosone/s/submit-now. Please also include the statement “There was no additional external funding received for this study.” in your updated Funding Statement.

Please include your amended Funding Statement within your cover letter. We will change the online submission form on your behalf.

A: *Funding Disclaimer: No additional external funding was received for this study, and all research was conducted on the following six topics.*

1. *2025 Guangdong Philosophy and Social Science Planning Project "Research on the Synergistic Evolutionary Mechanisms of the Governance System of Colleges and Universities and the Development of Students’ Socio-emotional Competence: Based on Multimodal Learning Analysis (GD25CJY29)".*
2. *2025 Key Research Project of Shenzhen Polytechnic University "Research on Key Methods for Analysis and Prediction of Social Behaviour of Specific Characters on Multimodal Big Data (6025310008K)".*
3. *2024 Guangdong Province Education Science Planning Project (Higher Education Special Project) "Research on Smart Classroom Teaching Behavior Analysis Methods Based on Scene Semantic Understanding and Deep Learning Characteristic Representation (2024GXJK766)".*
4. *2023 Guangdong Provincial Higher Vocational Education Teaching Reform Research and Practice Project "Research on Online Teaching Quality Evaluation Method Based on Multimodal Affective State Analysis (2023JG277)".*
5. *2024 Shenzhen Polytechnic University Quality Engineering Project "Research on Classroom Scene Understanding and Behavior Analysis Method Based on Multimodal Attention Mechanisms (7024310268)".*
6. *2024 Higher Education Scientific Research Planning Project of the Chinese Society of Higher Education "Research on the Analysis of Teaching and Learning Deep Interaction Characteristics in Smart Classroom Environment Supported by Multimodal Data (24XH0407)".*

5. We note that your Data Availability Statement is currently as follows: All relevant data are within the manuscript and its Supporting Information files.

Please confirm at this time whether or not your submission contains all raw data required to replicate the results of your study. Authors must share the “minimal data set” for their submission. PLOS defines the minimal data set to consist of the data required to replicate all study findings reported in the article, as well as related metadata and methods (https://journals.plos.org/plosone/s/data-availability#loc-minimal-data-set-definition).

For example, authors should submit the following data:

- The values behind the means, standard deviations and other measures reported;

- The values used to build graphs;

- The points extracted from images for analysis.

Authors do not need to submit their entire data set if only a portion of the data was used in the reported study.

If your submission does not contain these data, please either upload them as Supporting Information files or deposit them to a stable, public repository and provide us with the relevant URLs, DOIs, or accession numbers. For a list of recommended repositories, please see https://journals.plos.org/plosone/s/recommended-repositories.

If there are ethical or legal restrictions on sharing a de-identified data set, please explain them in detail (e.g., data contain potentially sensitive information, data are owned by a third-party organization, etc.) and who has imposed them (e.g., an ethics committee). Please also provide contact information for a data access committee, ethics committee, or other institutional body to which data requests may be sent. If data are owned by a third party, please indicate how others may request data access.

*A: The data and code applied in this paper uses only a portion of the data in the dataset, which can be accessed on request at https://github.com/hitlujin0808/education-Q-A*

6. PLOS requires an ORCID iD for the corresponding author in Editorial Manager on papers submitted after December 6th, 2016. Please ensure that you have an ORCID iD and that it is validated in Editorial Manager. To do this, go to ‘Update my Information’ (in the upper left-hand corner of the main menu), and click on the Fetch/Validate link next to the ORCID field. This will take you to the ORCID site and allow you to create a new iD or authenticate a pre-existing iD in Editorial Manager.

*A: The ORCIDs of the first and corresponding authors of this paper are registered, including the ORCID of JinLu (first author) is 0009-0004-5686-3654, and the ORCID of JiLi (corresponding author) is 0009-0003-3412-5493.*

7. Please ensure that you refer to Figure 1, 3, 7, 8 and 9 in your text as, if accepted, production will need this reference to link the reader to the figure.

*A: As revised, images 1, 3, 7, 8, and 9 are associated in the article with specific descriptions of their content.*

8. Please upload a copy of Figure 4, to which you refer in your text on page 19 in PDF submission. If the figure is no longer to be included as part of the submission please remove all reference to it within the text.

*A: As a result of the revision, figure 4 has been deleted and the presentation in the text has been adjusted and optimized accordingly.*

9. We note you have included a table to which you do not refer in the text of your manuscript. Please ensure that you refer to Table 1, 2, 3, 5, 6 and 7 in your text; if accepted, production will need this reference to link the reader to the Table.

*A: A total of seven tables are shown in this paper, and with some modifications, tables 1, 2, 3, 4, 5, 6, and 7 have been correlated in the text with corresponding descriptions.*

[Note: HTML markup is below. Please do not edit.]

Reviewers' comments:

Reviewer's Responses to Questions

Comments to the Author

1. Is the manuscript technically sound, and do the data support the conclusions?

The manuscript must describe a technically sound piece of scientific research with data that supports the conclusions. Experiments must have been conducted rigorously, with appropriate controls, replication, and sample sizes. The conclusions must be drawn appropriately based on the data presented.

Reviewer #1: Yes

Reviewer #2: Yes

2. Has the statistical analysis been performed appropriately and rigorously?

Reviewer #1: Yes

Reviewer #2: Yes

3. Have the authors made all data underlying the findings in their manuscript fully available?

The PLOS Data policy requires authors to make all data underlying the findings described in their manuscript fully available without restriction, with rare exception (please refer to the Data Availability Statement in the manuscript PDF file). The data should be provided as part of the manuscript or its supporting information, or deposited to a public repository. For example, in addition to summary statistics, the data points behind means, medians and variance measures should be available. If there are restrictions on publicly sharing data—e.g. participant privacy or use of data from a third party—those must be specified.

Reviewer #1: Yes

Reviewer #2: Yes

4. Is the manuscript presented in an intelligible fashion and written in standard English?

PLOS ONE does not copyedit accepted manuscripts, so the language in submitted articles must be clear, correct, and unambiguous. Any typographical or grammatical errors should be corrected at revision, so please note any specific errors here.

Reviewer #1: Yes

Reviewer #2: Yes

5. Review Comments to the Author

Please use the space provided to explain your answers to the questions above. You may also include additional comments for the author, including concerns about dual publication, research ethics, or publication ethics. (Please upload your review as an attachment if it exceeds 20,000 characters)

Reviewer #1: In summary, this article is interesting and offers valuable findings in terms of both theoretical knowledge and practical application. However, there are several weaknesses that need to be addressed to enhance the overall quality of the article. Language errors are evident, and the writing style lacks consistency, particularly in the labelling of tables and figures. Some labels use lowercase letters while others capitalize each word, which disrupts uniformity. Additionally, the labels for Figures 1, 2, and 3 are unclear, and the colours used are not appropriate for academic writing.

*A: Thank you for your valuable comments. Based on your suggestions, targeted changes have been made to the text as follows.*

1. *Grammatical issues in the text have been optimized, as detailed in red.*
2. *The formatting of all images and figure captions has been standardized in accordance with the journal's requirements.*
3. *Optimized description, title and colour scheme of images 1, 2, 3.*

The font used throughout the article is also inconsistent across the main text, references, and figure labels. For instance, the citation for “Figure 4” in the text does not follow a consistent formatting style. Moreover, it is recommended that Figure 4 be converted into a table, and all its content be incorporated accordingly for better clarity and presentation.

*A: As a result of the revision, figure 4 has been deleted and the presentation in the text has been adjusted and optimised accordingly.*

The presentation of research findings should also be further elaborated, with a more in-depth discussion that connects the current results to previous research findings and the theoretical framework underpinning the study.

*A: The presentation of the research results has been optimised and modified in conjunction with the structure of the paper, as detailed in Chapter 1 and the conclusions.*

Reviewer #2: Please consider correcting minor typographical errors such as “frameworkk” and “faik” to enhance the overall readability and polish of the manuscript.

*A: All of the above spelling issues have been corrected, and the article as a whole has also been grammar-checked to address all spelling errors.*

It may be helpful to clarify the distinctions between the different experimental configurations (e.g., RAG only, Code Interpreter only, and the combined approach) earlier in the methodology section to guide readers through the experimental design more clearly.

*A: Thanks for this valuable suggestion. In order to provide the reader with a clearer understanding of the comparative logic of the experimental design, a related section has been added at the beginning of the methodology section. Such a structural division will help the reader to establish a clear framework for comparison before delving into the details of the experiment. Subsequently, in the specific experimental descriptions, I will ensure the comparability between configurations through the control variable method (e.g., fixed pedestal model for GPT-4) and demonstrate the differences in the information processing paths of different configurations through flowcharts. This hierarchical description should effectively enhance the interpretability of the methodology.*

Including a visual system architecture diagram that illustrates the interaction between the RAG module, the Code Interpreter, and the LLM could significantly aid in understanding the proposed framework.

*A: The suggestions you have made are critical. In this paper, we have modified the contents of Figures 1, 2, and 3, especially in Figure 1, to implement the interaction between RAG module, code interpreter, and LLM that you proposed, i.e., RAG module output → context augmentation → LLM knowledge inference, for the whole research idea of the paper.*

To strengthen the rigor of the results, it is recommended to supplement the performance comparisons with appropriate statistical significance tests to demonstrate that the observed improvements are meaningful.

*A: Your suggestion of a statistical significance test is critical, which effectively validates the reliability of the experimental results, we have design a rigorous statistical analysis in the following ways, as in 5.6.5.*

*First, by applying statistical test methodology, it is determined that the differences in performance metrics between different configurations are caused by random factors.*

*Secondly, the performance of the conducted integrated approach is significantly better than the baseline configuration (one-sided test).*

*The detailed experimental results are detailed in Table 8.*

Providing additional detail on how the system handles or mitigates code execution errors—especially within the sandbox environment—would improve transparency and reproducibility.

*A: You have made a very good suggestion. In the article, we have added a multi-level error handling mechanism to ensure the reliability and reproducibility of code execution, as detailed in 4.10.*

*First, a scientific sandbox environment architecture was constructed to encapsulate the Python 3.10 runtime environment using Docker containers for containerised isolation.*

*Second, pre-checks are performed using AST parsing to carry out static pre-analysis and signal capture at the dynamic detection layer.*

*Third, in terms of error classification and processing, five cases are handled separately according to syntax errors, infinite loops, memory leaks, numerical computation errors, and missing third-party library dependencies.*

*Finally, the use of error type-guided correction is carried out to implement a self-correction strategy.*

It would be valuable to indicate whether any parameter tuning was performed for either the RAG or Code Interpreter components and, if so, describe the approach and settings used.

*A: Your question is the central detail of the experimental design of this paper. The full technical note on parameter tuning follows.*

*Your question is the central detail of the experimental design of this paper! A full technical note on parameter tuning follows.*

*First, the optimisation of the RAG component parameters is carried out. Performance improvements were achieved by fine-tuning BAAI/bge-reranker-large using LoRA and fine-tuning on a subset of MS MARCO Chinese.*

*Second, code interpreter parameter calibration is carried out. Optimise the execution environment configuration and control code generation.*

*Third, carry out collaborative control parameter tuning. According to the dynamic routing strategy, use the gated attention mechanism to carry out information fusion strategy adjustment, and on the MultiArith dataset, achieve fusion accuracy improvement.*

Expanding on the use of Chain-of-Thought prompting with a concrete example could help readers better appreciate how this strategy supports multi-step reasoning within the system.

*A: Your question is a very good one, and we have added a case study in Chapter 5. In the following, the mechanism of Chain-of-Thought (CoT) hints operating in the system of this paper is disassembled in detail through a specific case of Multi-Modal reasoning.*

*Given that a teaching building has four floors, each with east and west stairs, the number of different ways to go from the first floor to the fourth floor is ( ).*

*A.7 B.8 C.9D.10*

*Answer: Let's think step by step. According to the problem, the teaching building has four floors, and each floor has east and west stairs. There are2 ways to go from the first floor to the second floor, similarly from the second floor to the third floor, and from the third floor to the fourth floor, each having 2 ways. Therefore, the total number of ways from the first floor to the fourth floor is 2 x 2x 2 = 8,so the answer is B.*

*This case shows how the system achieves a verifiable reasoning process through problem decomposition, knowledge retrieval, procedural verification, and multi-source alignment.*

The error analysis section would benefit from the inclusion of illustrative examples of failure cases, along with brief suggestions or hypotheses about how such challenges might be addressed in future work.

*A: Your suggestions are very good! The following typical failures encountered in stress testing our system and their in-depth analyses have been added to 5.6.5.*

*Case Name: Time-sensitive knowledge retrieval failure.*

*Question input: What is the length of the context window for OpenAI's newly released GPT-5 model?*

*Error output: The GPT-5 supports a context window of 128k tokens.*

*Problem description: Actual fictitious data, the real situation is not published.*

*Cause Analysis:*

*1.RAG module retrieved outdated technology blogs (December 2023) .*

*2. Knowledge base update is delayed (latest update is January 2024) .*

*3. Temporal context extraction failed: ‘Latest’ was not correctly mapped to the temporal filter.*

*Improvement measures:*

*Improvements: access to real-time data APIs (e.g., Google Alerts) and add a time decay factor to the vector index.*

A brief discussion of potential ethical concerns or risks related to the deployment of large language models in educational contexts—such as bias, misinformation, or over-reliance—could add depth to the manuscript.

*A: Your suggestion is excellent, and the material related to the ethical issues or risks associated with deploying large-scale language models in educational settings has been investigated and increased to chapter 6.5.*

*The deployment of LLM in educational environments, despite their great potential is accompanied by significant ethical risks and challenges. These risks may not only affect student learning outcomes, but may also have far-reaching implications for educational equity, cognitive development and even social values. First, there is the problem of bias due to data bias. Large-scale language models are trained based on a large amount of data, and if there are biases in these data, such as over-representation or under-representation of certain groups of people, one-sided emphasis on certain points of view, etc., then the model may learn these biases and then show bias towards specific groups and ideas in the generated content, which may adversely affect the students' values and reinforce their stereotypes of certain groups of people Impression. Second, the problem of bias in the algorithm itself, the algorithmic structure and design of the language model may also introduce bias. For example, the attention mechanism of the model may unconsciously give higher weight to certain types of information and relatively ignore others, leading to unfairness in the generated results. Such algorithmic biases may not be easily detected, but can subconsciously affect information transfer and student cognitive formation in educational settings. Third, inertia of students' thinking. If students rely excessively on large-scale language models in the learning process and seek answers from the models directly when they encounter problems instead of thinking, analyzing and exploring on their own, they will gradually develop thinking inertia, which is not conducive to the development of their critical thinking, creativity and problem-solving skills. In the long run, this will affect students' overall quality and future development. Finally, educator dependency: educators may also rely excessively on large-scale language models for teaching, for example, by directly using model-generated lesson plans, teaching content, and so on, while neglecting their own professional knowledge and teaching experience. This may lead to a decline in the quality of teaching and a limitation in the professional development of educators, as well as an inability to tailor teaching to the actual situation of students.*

Lastly, while the data sources are clearly indicated, providing access to any code or implementation details (e.g., via a GitHub repository) would further support reproducibility and allow others to build upon this valuable work.

A: *The data and code applied in this paper uses only a portion of the data in the dataset, which can be accessed on request at https://github.com/hitlujin0808/education-Q-A.*

6. PLOS authors have the option to publish the peer review history of their article (what does this mean?). If published, this will include your full peer review and any attached files.

If you choose “no”, your identity will remain anonymous but your review may still be made public.

Do you want your identity to be public for this peer review? For information about this choice, including consent withdrawal, please see our Privacy Policy.

Reviewer #1: No

Reviewer #2: Yes: Muralidhar Kurni, Ph.D., PostDoc
