# Supplementary material for: A novel framework for educational Q&A: Leveraging RAG and Code Interpreters for knowledge retrieval and logical computation
Source: PLoS One. 2025 Dec 1;20(12):e0337361. doi: 10.1371/journal.pone.0337361 (PMC12668533; doi:10.1371/journal.pone.0337361)
Supplement: Attachment — Submitted filename: Response to Reviewers.docx [file pone.0337361.s007.docx]

Journal Requirements:

1. If the reviewer comments include a recommendation to cite specific previously published works, please review and evaluate these publications to determine whether they are relevant and should be cited. There is no requirement to cite these works unless the editor has indicated otherwise.

***A：*** We will carry out the paper revision work in strict accordance with the requirements.

2. Please review your reference list to ensure that it is complete and correct. If you have cited papers that have been retracted, please include the rationale for doing so in the manuscript text, or remove these references and replace them with relevant current references. Any changes to the reference list should be mentioned in the rebuttal letter that accompanies your revised manuscript. If you need to cite a retracted article, indicate the article’s retracted status in the References list and also include a citation and full reference for the retraction notice.

***A：*** Some references in the references have been replaced according to the requirements of the journal to enhance the accuracy of the citation. In addition, the DOI of the arXiv paper is supplemented to facilitate the paper query.

[Note: HTML markup is below. Please do not edit.]

Reviewers' comments:

Reviewer's Responses to Questions

Comments to the Author

1. If the authors have adequately addressed your comments raised in a previous round of review and you feel that this manuscript is now acceptable for publication, you may indicate that here to bypass the “Comments to the Author” section, enter your conflict of interest statement in the “Confidential to Editor” section, and submit your "Accept" recommendation.

Reviewer #1: All comments have been addressed

Reviewer #2: All comments have been addressed

***A：*** We would like to express our gratitude to the reviewers for their hard work.

2. Is the manuscript technically sound, and do the data support the conclusions?

The manuscript must describe a technically sound piece of scientific research with data that supports the conclusions. Experiments must have been conducted rigorously, with appropriate controls, replication, and sample sizes. The conclusions must be drawn appropriately based on the data presented.

Reviewer #1: Yes

Reviewer #2: Yes

***A：*** We would like to express our gratitude to the reviewers for their hard work.

3. Has the statistical analysis been performed appropriately and rigorously?

Reviewer #1: Yes

Reviewer #2: (No Response)

***A：*** We would like to express our gratitude to the reviewers for their hard work.

4. Have the authors made all data underlying the findings in their manuscript fully available?

The PLOS Data policy requires authors to make all data underlying the findings described in their manuscript fully available without restriction, with rare exception (please refer to the Data Availability Statement in the manuscript PDF file). The data should be provided as part of the manuscript or its supporting information, or deposited to a public repository. For example, in addition to summary statistics, the data points behind means, medians and variance measures should be available. If there are restrictions on publicly sharing data—e.g. participant privacy or use of data from a third party—those must be specified.

Reviewer #1: Yes

Reviewer #2: Yes

***A：*** We would like to express our gratitude to the reviewers for their hard work.

5. Is the manuscript presented in an intelligible fashion and written in standard English?

PLOS ONE does not copyedit accepted manuscripts, so the language in submitted articles must be clear, correct, and unambiguous. Any typographical or grammatical errors should be corrected at revision, so please note any specific errors here.

Reviewer #1: Yes

Reviewer #2: Yes

***A：*** We would like to express our gratitude to the reviewers for their hard work.

6. Review Comments to the Author

Please use the space provided to explain your answers to the questions above. You may also include additional comments for the author, including concerns about dual publication, research ethics, or publication ethics. (Please upload your review as an attachment if it exceeds 20,000 characters)

Reviewer #1: In general, the author has addressed the comments and made appropriate improvements accordingly. There is a noticeable improvement in the overall quality of the writing, reflecting the author’s effort to enhance clarity and coherence

***A：***Thank you again for your hard work, and we will continue to learn your valuable opinions and improve the quality of the paper.

Reviewer #2: The revised manuscript demonstrates significant improvements and adequately addresses all comments raised in the previous review round. The integration of Retrieval-Augmented Generation (RAG) and LLM-based Code Interpreters is well-motivated and technically justified. The experimental design is clear, and the four comparative configurations are now explicitly outlined early in the methodology section, enhancing reader comprehension.

***A：***Thank you again for your hard work, and we will continue to learn your valuable opinions and improve the quality of the paper.

The addition of the system architecture diagram (Figure 1) helps visualize the interaction between RAG, the Code Interpreter, and the LLM, which strengthens the conceptual clarity. The statistical significance tests introduced in Section 5.6.5 provide rigor to the performance claims, and the results in Table 8 confirm the robustness of the findings.

***A：***Thank you again for your hard work, and we will continue to learn your valuable opinions and improve the quality of the paper.

The authors have also improved reproducibility and transparency by sharing the implementation code via GitHub and including a detailed discussion of error handling within the sandboxed execution environment. The inclusion of a Chain-of-Thought example and illustrative failure cases further enriches the manuscript, offering practical insights into system behavior.

***A：***Thank you again for your hard work, and we will continue to learn your valuable opinions and improve the quality of the paper.

Finally, the newly added discussion on ethical concerns, covering bias, misinformation, and over-reliance, adds valuable depth and relevance, especially for educational applications of LLMs.

***A：***Thank you again for your hard work, and we will continue to learn your valuable opinions and improve the quality of the paper.

The manuscript is now clearly written, technically sound, and methodologically rigorous. I believe it is suitable for publication in its current form.

***A：***Thank you again for your hard work, and we will continue to learn your valuable opinions and improve the quality of the paper.

7. PLOS authors have the option to publish the peer review history of their article (what does this mean?). If published, this will include your full peer review and any attached files.

If you choose “no”, your identity will remain anonymous but your review may still be made public.

Do you want your identity to be public for this peer review? For information about this choice, including consent withdrawal, please see our Privacy Policy.

Reviewer #1: No

Reviewer #2: Yes: Muralidhar Kurni, Ph.D., PostDoc.

***A：*** We would like to express our gratitude to the reviewers for their hard work.
