# Supplementary material for: A novel framework for educational Q&A: Leveraging RAG and Code Interpreters for knowledge retrieval and logical computation
Source: PLoS One. 2025 Dec 1;20(12):e0337361. doi: 10.1371/journal.pone.0337361 (PMC12668533; doi:10.1371/journal.pone.0337361)
Supplement: Attachment — Submitted filename: 20251024Response of Reviewer(Plos one).doc [file pone.0337361.s008.doc]

Journal Requirements:

If the reviewer comments include a recommendation to cite specific previously published works, please review and evaluate these publications to determine whether they are relevant and should be cited. There is no requirement to cite these works unless the editor has indicated otherwise.

*A：Thank you for your suggestions. I shall carefully review the reviewers' comments regarding the references and promptly make the necessary improvements.*

Please review your reference list to ensure that it is complete and correct. If you have cited papers that have been retracted, please include the rationale for doing so in the manuscript text, or remove these references and replace them with relevant current references. Any changes to the reference list should be mentioned in the rebuttal letter that accompanies your revised manuscript. If you need to cite a retracted article, indicate the article’s retracted status in the References list and also include a citation and full reference for the retraction notice.

*A：Thank you for your suggestion. All references have been checked and are indeed articles from the database.*

Comments from the Editorial Office:

In the previous decision letter, the PLOS One Editorial Office requested that you address the following points, which remain unaddressed. Please give further attention to these requirements before submitting a revised manuscript and response document:

1) We thank you for providing a link to the repository from which the code underpinning this study can be freely accessed (https://github.com/hitlujin0808/education-Q-A). We note that this link has been provided in your Response to Reviewers file, but does not appear to have been included in your manuscript text. Please revise your manuscript text to include this link.

*A：Thank you for your suggestion. The code link has been added at the end of the paper.*

2) We note that the documentation for the code in the repository you have linked is provided in a language other than English. Because PLOS One is an international journal published in English, we kindly ask that you update the documentation in this repository to include translation into English. This is to ensure that your study complies with the PLOS One policy on code sharing, which states that you are encouraged to share your code 'in a way that follows best practice and facilitates reproducibility and reuse' (https://journals.plos.org/plosone/s/materials-software-and-code-sharing#loc-sharing-code).

*A：Thank you for your suggestion. The explanatory notes for the code have now been fully converted into English.*

[Note: HTML markup is below. Please do not edit.]

Reviewers' comments:

Reviewer's Responses to Questions

Comments to the Author

1. If the authors have adequately addressed your comments raised in a previous round of review and you feel that this manuscript is now acceptable for publication, you may indicate that here to bypass the “Comments to the Author” section, enter your conflict of interest statement in the “Confidential to Editor” section, and submit your "Accept" recommendation.

Reviewer #1: All comments have been addressed

*A：Thank you for your support.*

Reviewer #2: All comments have been addressed

*A：Thank you for your support.*

2. Is the manuscript technically sound, and do the data support the conclusions?

The manuscript must describe a technically sound piece of scientific research with data that supports the conclusions. Experiments must have been conducted rigorously, with appropriate controls, replication, and sample sizes. The conclusions must be drawn appropriately based on the data presented.

Reviewer #1: Yes

*A：Thank you for your support.*

Reviewer #2: Yes

*A：Thank you for your support.*

3. Has the statistical analysis been performed appropriately and rigorously?

Reviewer #1: Yes

*A：Thank you for your support.*

Reviewer #2: Yes

*A：Thank you for your support.*

4. Have the authors made all data underlying the findings in their manuscript fully available?

The PLOS Data policy requires authors to make all data underlying the findings described in their manuscript fully available without restriction, with rare exception (please refer to the Data Availability Statement in the manuscript PDF file). The data should be provided as part of the manuscript or its supporting information, or deposited to a public repository. For example, in addition to summary statistics, the data points behind means, medians and variance measures should be available. If there are restrictions on publicly sharing data—e.g. participant privacy or use of data from a third party—those must be specified.

Reviewer #1: (No Response)

*A：Thank you for your support.*

Reviewer #2: Yes

*A：Thank you for your support.*

5. Is the manuscript presented in an intelligible fashion and written in standard English?

PLOS ONE does not copyedit accepted manuscripts, so the language in submitted articles must be clear, correct, and unambiguous. Any typographical or grammatical errors should be corrected at revision, so please note any specific errors here.

Reviewer #1: (No Response)

*A：Thank you for your support.*

Reviewer #2: Yes

*A：Thank you for your support.*

6. Review Comments to the Author

Please use the space provided to explain your answers to the questions above. You may also include additional comments for the author, including concerns about dual publication, research ethics, or publication ethics. (Please upload your review as an attachment if it exceeds 20,000 characters)

Reviewer #1: COMMENTS.

SO FAR ONLY THOSE COMMENTS HAVE SYSTEM ERROR!

PAGE 11_ 1. Introduction

The educational question-answering systemError! Reference source not found. is playing an increasingly important role in modern education.

*A：Thank you for your suggestion. The issue has been resolved by replacing the reference 1&2.*

PAGE 12_ However, despite their excellence in text generation and comprehension, LLMs still face limitations in handling complex reasoning and mathematical calculations, and they are susceptible to the “hallucination[8]” problem, where the model may generate incorrect answers that do not align with actual knowledge, posing challenges to the accuracy and reliability of the educational systemError! Reference source not found..

*A：Thank you for your suggestion. The issue has been resolved by replacing the reference 8.*

To address these challenges in current educational question-answering systems, this study proposes a new LLM-based educational question-answering system that combines RetrievalAugmented Generation[9][11]Error! Reference source not found. (RAG) and LLM Code Interpreter[12][14][15][16].

*A：Thank you for your suggestion. The issue has been resolved by replacing the reference 10&11.*

PAGES 14_ 2 Related works

2.1 Knowledge Base Question Answering Systems (KBQA)

Past educational question answering systems primarily relied on Knowledge Base Question Answering Systems[20][21]Error! Reference source not found.

*A：Thank you for your suggestion. The issue has been resolved by replacing the reference 20&21.*

PAGES15_ Second, methods based on structured knowledge bases still face considerable challenges in dealing with the diversity and complexity of natural languageError! Reference source not found..

*A：Thank you for your suggestion. The issue has been resolved by replacing the reference 22.*

PAGES 16_ it may still generate inaccurate or erroneous results when confronted with tasks that require multi-step calculations or precise answersError! Reference source not found..

*A：Thank you for your suggestion. The issue has been resolved by replacing the reference 32.*

PAGES 16_2.3 Large Language Models (LLMs) and Code Interpreters

To address the limitations of LLMs in reasoning and computation, researchers have gradually introduced the Code Interpreter component[16][32]. A Code Interpreter is a tool that can generate and execute code, allowing it to handle complex tasks such as mathematical operations[34], data manipulation[35], and logical reasoningError! Reference source not found.

*A：Thank you for your suggestion. The issue has been resolved by replacing the reference 36.*

PAGES 18_ 4. Textbook Question Answering (TQA)

The TQAError! Reference source not found. dataset comes from high school science textbooks and includes 1,076 course contents from life sciences, Earth sciences, and physical sciences.

*A：Thank you for your suggestion. The issue has been resolved by replacing the reference 41.*

PAGES 21_ 4.4 Implementation Details of the RAG Retrieval Module

The RAG mechanism was implemented using the following approach, including knowledge base construction, retrieval and text selection, as well as fusion and input. The knowledge base was constructed by integrating publicly available text sources, such as Wikipedia excerpts, popular science websites, and digitized versions of various textbooks in both Chinese and English to ensure

linguistic consistency in retrieval. The original text was segmented into sentences or paragraphs, and vector-based retrieval techniquesError! Reference source not found. (e.g., Faiss or Milvus) were used to index the text

*A：Thank you for your suggestion. The issue has been resolved by replacing the reference 46.*

PAGES 22_ 4.6 Prompt Engineering

To maximize the synergistic effects of the LLM, RAG, and Code Interpreter components, careful prompt engineering techniques[44][45]Error! Reference source not found. were employed.

*A：Thank you for your suggestion. The issue has been resolved by replacing the reference 45&46&49.*

Reviewer #2: The manuscript has undergone substantial improvement in both clarity and methodological rigor compared to earlier versions.

*A：Thank you for your support.*

The integration of Retrieval-Augmented Generation (RAG) and LLM-based Code Interpreters is well-motivated, technically justified, and convincingly demonstrated across diverse educational datasets.

*A：Thank you for your support.*

The addition of the system architecture diagram, statistical significance testing, and error analysis has strengthened the scientific quality and credibility of the work.

*A：Thank you for your support.*

Sharing the implementation code via GitHub enhances transparency, reproducibility, and the practical applicability of the study.

*A：Thank you for your support.*

The discussion of ethical considerations—bias, misinformation, and over-reliance—is highly relevant and adds value to the paper, especially in educational contexts.

*A：Thank you for your support.*

The writing is clear, coherent, and presented in standard English, making the manuscript accessible to a broad readership.

*A：Thank you for your support.*

Overall, the research is technically sound, the data support the conclusions, and the paper is suitable for publication in its current form.

*A：Thank you for your support.*

7. PLOS authors have the option to publish the peer review history of their article (what does this mean?). If published, this will include your full peer review and any attached files.

If you choose “no”, your identity will remain anonymous but your review may still be made public.

Do you want your identity to be public for this peer review? For information about this choice, including consent withdrawal, please see our Privacy Policy.

Reviewer #1: No

*A：Thank you for your support.*

Reviewer #2: Yes: Muralidhar Kurni

*A：Thank you for your support.*
